# Assessment of the Construction of a Climate Resilient City: An Empirical Study Based on the Difference in Differences Model

**DOI:** 10.3390/ijerph18042082

**Published:** 2021-02-21

**Authors:** Zifeng Liang

**Affiliations:** School of Public Policy & Management, Tsinghua University, Beijing 100084, China; liangzf19@mails.tsinghua.edu.cn

**Keywords:** climate resilient cities, multi-level grey system evaluation method, assessment, difference in differences model

## Abstract

Facing climate risks has become a common problem for mankind and a topic of great importance for the Chinese government. To thoroughly implement the overall requirements for the construction of an ecological civilization and effectively improve the capacity of cities to adapt to climate change, China launched the pilot construction of “Climate Resilient Cities” in 2017. In this paper, 16 prefecture level cities in Anhui Province of China were selected as the research objects, and the multi-level grey system evaluation method was used to measure the climate resilience of these regions. We used the difference in differences method to evaluate the effect of the pilot policy of “Climate Resilient Cities.” The pilot policies of the “Climate Resilient Cities” showed a significant contribution to the regional climate resilience, and, after isolating the impact of other factors on the regional climate resilience, the pilot policies of the “Climate Resilient Cities” increased the climate resilience of the pilot cities by four percentage points. The pilot policies of the “Climate Resilient Cities” had a significant contribution to the urban infrastructure development and ecological space optimization, as well as non-significant impacts to the urban water security, emergency management capacity-building, and science and technology innovation initiatives.

## 1. Introduction

The report Human Costs of Disasters 2000–2019, published on 12 October 2020 by the United Nations Office for Disaster Risk Reduction (UNISDR) and the Centre for Research on the Epidemiology of Disasters of the University of Leuven, Belgium, states that “Between 2000 and 2019, 7348 natural disasters were recorded globally, killing 1.23 million people, affecting a total of 4 billion people, and causing $2.97 trillion in global economic losses. The global total number of natural disasters has risen dramatically in the first two decades of the twenty-first century, with an alarming increase in the number of climate-related disasters in particular,” and “the surge in the number of climate-related disasters has been a major factor in the rise in the total number of disasters.” This series of natural disasters is a constant reminder that climate change has become a common challenge for mankind, and that addressing the risks posed by climate change should be a common task for all of us [1].

The United Nations attaches great importance to the risks posed by climate change to measure its effects in a more scientific manner. The United Nations Environment Programme (UNEP) and the World Meteorological Organization jointly established the United Nations intergovernmental panel on climate change (IPCC), which was released by the Commission in 2013. The Fifth Assessment Report stated that, “climate change is real and human activity is the primary cause of its occurrence.” It is thus clear that global climate change has become a major challenge for all countries, and there is broad international consensus that we should work together to combat it.

China signed the United Nations Framework Convention on Climate Change (UNFCCC) as early as 1992 at the Earth Summit convened by the United Nations. At the level of laws and regulations, the Chinese government has successively promulgated a series of laws, regulations, and policies on the prevention and control of air pollution, clean production, energy conservation, and emission reduction. At the organizational level, to address climate change in a more targeted manner, China established “the National Leading Group on Climate Change, Energy Conservation and Emission Reduction” in June 2007 as the national coordinating body for climate change, energy conservation, and emission reduction, with the Ministry of Ecology and Environment and the Development and Reform Commission assuming specific responsibilities; at the practical level. To implement actions to address climate change, the Chinese government has carried out a series of pilot construction projects in recent years, including low-carbon cities and sponge cities, and has achieved results. In February 2016, the National Development and Reform Commission (NDRC), the department of housing and urban–rural development led initiatives to develop the city to adapt to the climate change action plan. In its overall requirements, the program referred to “strengthening scientific and technological support, firmly establishing the concept of adaptation, comprehensively promoting urban adaptation to climate change from the aspects of policies and regulations, institutional mechanisms, overall planning, standards and norms, construction, and management; striving to create climate-adaptive cities; and comprehensively enhancing urban resilience to climate change.”

The National Development and Reform Commission (NDRC) and the Ministry of Housing and Construction (MOHURD) issued the Pilot Program for the Construction of Climate-Resilient Cities in August 2016 to encourage cities to actively apply. In February 2017, the National Development and Reform Commission (NDRC) and the Ministry of Housing and Urban–Rural Development (MOHURD) issued the Notice of Pilot Work on the Construction of Climate Resilient Cities, which included 28 cities, including Hefei City and Huaibei City in Anhui Province, Haikou City in Hainan Province, and Guangyuan City in Sichuan Province, on the pilot list of “Climate Resilient Cities,” marking the beginning of China’s attempt to progressively explore the full range of cities’ climate risk management capacity from a practical level by means of policy pilots.

2020 is a key year for the pilot construction of Climate Resilient Cities, and the Notice on Pilot Construction of Climate Resilient Cities clearly states that “By 2020, the infrastructure for adapting to climate change in pilot areas will be strengthened, the adaptive capacity will be significantly improved, public awareness will be significantly enhanced, a number of typical example cities with international advanced level will be built, and a series of replicable and scalable pilot experience will be formed.” Thus far, the policy has been implemented for four years; however, there is a lack of scientific answers to questions, such as how the policy has been implemented so far, what experiences and shortcomings exist, and what kind of improvements need to be made in the next step, and the answers to these questions are not only of great practical significance for the next step in the construction of Climate Resilient Cities, but also of great theoretical significance for the study of how cities can cope with climate change. Although the concept of climate resilient city has been widely concerned by scholars and policymakers, there is still a lack of theoretical and empirical research in this field, especially the lack of evaluation on the construction status of climate resilient city. The purpose of this paper is: First, to evaluate the construction of climate resilient cities by establishing a more perfect evaluation index system; second, to comprehensively calculate the index system through appropriate econometric methods, so as to achieve scientific and effective policy evaluation.

Scholars in different fields have carried out extensive research on climate resilient cities. The current research on Climate Resilient Cities focuses on three main areas: the characteristics of Climate Resilient Cities, how to plan and build Climate Resilient Cities, and the assessment of the construction of Climate Resilient Cities. At present, the concept and characteristics of climate resilient cities are clearly defined [2,3,4], and how to plan and build climate resilient cities is also studied from the perspective of urban planning, disaster prevention and control [5,6,7,8]. However, limited by data and analysis methods, few scholars evaluate the construction of climate resilient cities, especially the quantitative evaluation. This paper mainly focuses on how to evaluate the construction of climate resilient cities. The main difference between this paper and previous studies is that this paper adopts the method of empirical research, constructs a set of operable evaluation system through the multi-level grey system evaluation method, and realizes the evaluation of the construction of climate resilient cities through the difference in differences model.

The theoretical contributions of this paper are as follows: first, a complete assessment index of the construction of Climate Resilient Cities is constructed, and all the indexes are quantified, which improves the quantitative research of Climate Resilient Cities. Second, the multi-level grey system evaluation method is introduced into the research of Climate Resilient Cities, which effectively solves the problem of “small sample and poor information” and realizes a more scientific and effective assessment of the construction of Climate Resilient Cities. Third, the effect of the construction of Climate Resilient Cities is more clearly identified by using the difference in differences (DID) model.

This paper is organized in the following manner. Section 2 provides a literature review; Section 3 describes the data sources and how the variables were measured; Section 4 describes the setup of the difference in differences model and the applicability test; Section 5 presents the regression model and regression results; and Section 6 and Section 7 present the discussion and conclusions, respectively.

## 2. Literature Review

The concept of resilience, since the nineteenth century, has gradually been applied to mechanics, physics [9], psychology [10,11], and different fields of study. The concept of resilience was first introduced to the field of systems ecology by the Canadian ecologist Holling to define the characteristics of a steady state ecosystem. Since the 1990s, scholars have gradually extended the study of resilience from natural ecology to human ecology [12]. The concept of resilience has undergone many evolutions, from the initial engineering resilience to ecological resilience and finally to evolutionary resilience. Holling defines resilience as “engineering resilience,” which is the ability of a system to return to an equilibrium or stable state after the application of a disturbance [6]. Holling then revised his definition of resilience and introduced the concept of “ecological resilience,” which he defined as “the magnitude of the disturbance that a system can absorb before changing its own structure” [13]. Walker introduced the concept of “evolutionary resilience,” defining resilience as “the ability of complex social-ecological systems to stimulate change, adaptation and transformation in response to stresses and constraints” [14].

As an essential research subject of human ecology, the idea of resilience is naturally applied to urban research, laying the intellectual foundation for the formation of the tough city theory [15]. “The concept of a “Resilient City” has been widely appreciated by academics and governments since the first International Conference on Cities and Adaptation to Climate Change, convened by the United Nations Committee on Disaster Reduction in Bonn. For example, in 2002, the UK launched ”the UK Climate Impact Programme”, which aimed to provide scientific support for climate policymaking in the UK; and, in 2008, the American Planning Association published the Planning and Climate Change Policy Guide, which suggested that urban planning can play an active role in addressing climate change risks through policy and methodological innovation, providing a reference for policymakers [16].

Cities, such as Cape Town, South Africa; Toronto, Canada; and Copenhagen, Denmark, have also formulated and implemented plans to cope with climate change and improve their climate adaptation capacity, accumulating valuable experience for the construction of Climate Resilient Cities. In addition, the Making Cities Resilient campaign of the United Nations International Strategy for Disaster Reduction (UNISDR) and the 100 Resilient Cities initiative of the Rockefeller Foundation are both positive examples of efforts to improve the climate resilience of cities. As resilient cities are primarily responses to climate change, they are also referred to as “Climate Resilient Cities” in many studies and national policy texts, and for ease of understanding, the terms “Resilient City” and “Climate Resilient City” are referred to collectively as “Climate Resilient City” in the following text.

The current research on Climate Resilient Cities focuses on three main areas: the characteristics of Climate Resilient Cities, how to plan and build Climate Resilient Cities, and the assessment of the construction of Climate Resilient Cities. Representative scholars in the field of “Concepts and characteristics of Climate Resilient Cities” include Godschalk, Campanella, and Jha, who defined the characteristics of Climate Resilient Cities in detail. Physical systems are the constructed and natural environmental components of the city. They include its built roads, buildings, infrastructure, communications, and energy facilities, as well as its waterways, soils, topography, geology, and other natural systems. The communities act as the brain of the city, directing its activities, responding to its needs, and learning from its experience [2]. Campanella places great emphasis on the role of human communities, noting that politics, economics, and citizenship are all important factors in urban resilience, with Campanella highlighting that “Urban resilience is largely a function of resilient and resourceful citizens” [3]. Jha, Miner, and Stanton further identified four main components of urban resilience, namely, infrastructural resilience, institutional resilience, economic resilience, and social resilience [4]. Infrastructural resilience refers to the reduction of vulnerability of built structures and facilities, and also encompasses the accessibility of lifeline projects and the emergency response capacity of urban communities; institutional resilience focuses on the ability of governmental and non-governmental organizations to guide communities in their governance; economic resilience refers to the economic diversity of urban communities to be able to respond to crises; and social resilience is seen as the integration of elements, such as the demographic characteristics of urban communities, the way they are organized, and their human capital [8].

However, currently, there is still a lack of a unified standard for the identification of Climate Resilient Cities. The author believes that Climate Resilient Cities are cities that have incorporated the concept of climate change adaptation into the whole process of urban planning and construction management, improved the relevant planning and construction standards and systems, strengthened the infrastructure for climate change adaptation, improved the awareness of urban residents of protection, and, thus, significantly improved their ability to adapt to climate change and their ability to resist risks.

Representative scholars in the field of “How to plan and build Climate Resilient Cities” include Pickett, Vale, and Ahern. Vale, Campanella, and Thomas were the first to suggest in 2005 that “building resilience capacity through landscape and urban planning requires that planners and designers identify the stochastic processes and disturbances that a particular landscape or city is likely to face, the frequency and intensity of these events, and how cities can build the adaptive capacity to respond to these disturbances while remaining in a functional state of resilience” [5]. Pickett, Cadenassso, Grove, and others argued that it is important to focus on “the role of spatial heterogeneity in both the ecological and social functioning of urban areas and the consequent perspective of metropolitan areas as integrated ecological–social systems,” in addition to proposing three ways to enhance the urban heterogeneity. The first is the recognition of a “learning loop” in metropolitan ecosystems, the second is the use of urban design as experiments whose ecological and social outcomes can be measured, and finally is the potency of a dialog between professionals and citizens, communities, and institutions to support both research and design [6].

Based on the synthesis of previous studies, Ahern proposed that, “A proposed suite of five urban planning and design strategies for building urban resilience includes: multifunctionality, redundancy and modularization, (bio and social) diversity, multi-scale networks and connectivity, and adaptive planning and design [7]” On the basis of the analysis of 28 pilot programs for Climate Resilient Cities in China, Li Huimin, Qiu Ping, and others indicated that scientific assessment, focus, and sharing of results should be achieved in climate risk assessment; the system should be formulated in a way that integrates various types of risks, multi-sectoral coordination, diversified means, long-term planning, consideration of the output ratios and measurable effects; and the government, enterprises, the public, and other organizations should be jointly involved in the construction, with diversified means of governance and timely feedback [8]. 

Zheng Yan, Zhai Jianqing, Wu Zhangyun, and other scholars advocated strengthening the synergistic construction of Climate Resilient Cities and sponge cities, constructing scientific and feasible assessment indexes for the classification of resilient cities, adopting differentiated policy support for different types of pilot cities, and strengthening public participation in the construction of resilient cities [17]. These studies are informing the national decision-making on Climate Resilient Cities; however, countries must still tailor their policies to the local conditions. Therefore, it is necessary to establish a set of more perfect assessment indicators of Climate Resilient Cities’ construction. However, the existing research on the indicator system is not enough, so this study intends to establish a set of more perfect and more applicable indicator system.

Research on the characteristics, planning, and construction of Climate Resilient Cities is well established, but there is still a lack of quantitative assessment of the state of the construction of Climate Resilient Cities. Scientific and effective evaluation not only enables policymakers to understand the current situation of Climate Resilient Cities, but also facilitates further work; thus, policy evaluation of Climate Resilient Cities is particularly important. Kakimoto compared the local hazard mitigation plans of three developed countries (the USA, Japan, and Korea) in the process of building Climate Resilient Cities, and then analyzed the construction and use of flood control facilities in the process of building Climate Resilient Cities by establishing an index system, and made corresponding policy recommendations [18].HaunJung, SeEun, Shin, etc., evaluated and analyzed disasters, accidents, factors that threaten cities, and elements of the policy environment based on the evaluation system proposed by “100 Resilient Cities” and made policy recommendations on urban safety and the policy environment in Seoul based on the results of the study [19]. Xie Xinlu and Zheng Yan analyzed the climate resilience, economic support capacity, social development capacity, natural resource endowment, technological adaptation capacity, and risk management capacity of each district in Beijing, China from 2010 to 2014, and pointed out that the construction of Climate Resilient Cities should reflect the leading role of the government and promote the collaborative development of different spatial regions through forward-looking adaptation planning to enhance the overall adaptive capacity and resilience of the entire urban system [20]. Fitzgibbons and Mitchel used a sample of 31 of the 100 Climate Resilient Cities selected by the Rockefeller Foundation to assess the cities’ climate resilience and to highlight issues of equity and justice in the process of building Climate Resilient Cities [21,22]. Wu Bohong and Chen An studied the resilience evaluation model of Climate Resilient Cities, analyzed the evolution mechanisms of different stages of Climate Resilient Cities in the process of encountering external disturbances, and proposed the social significance of key nodes in the evolution of Climate Resilient Cities’ resilience, providing a quantitative evaluation index reference for the long-term adaptive capacity and wisdom of cities [23]. The ability of cities to adapt to climate is closely related to climate conditions; however, the natural recovery cycle is very long, and without government policy support and people’s joint efforts, it is difficult to recover the environmental losses caused by human activities in a short period of time. Thus, the evaluation of policies for building climate-resilient cities has strong practical significance for the construction of Climate Resilient Cities. Although the aforementioned scholars have conducted preliminary explorations of Climate Resilient Cities, the field still has areas where the research can be expanded.

The above-mentioned studies often evaluate the construction of Climate Resilient Cities from microscopic perspectives, such as the city’s flood control capacity and ecological nourishment capacity, which cannot fully reflect the overall situation of Climate Resilient Cities. In addition, the measurement methods of these studies are relatively simple, which makes it difficult to clearly identify the effects of climate-resilient city-related policies, and these studies can only prove that the climate resilience of cities does change after the national policies are promulgated; however, they cannot prove that this change is caused by the policies, because the climate resilience of a city will still change even if the government does not promulgate the policies. Therefore, scientific and reasonable econometric methods must be used to clearly identify the effects of Climate Resilient Cities pilot policies [24].

The difference in differences method can eliminate the factors that may affect the policy effect through calculation, therefore, it is one of the most important methods in the field of policy evaluation research and is widely used in various policy evaluation processes. Some of the more representative ones include Orley Ashenfelter, who first introduced the difference in differences method into the social sciences in 1978, studying the effect of training programs on earnings [25]. Eissa and Liebman, 1996, demonstrated through the difference in differences method that the 1986 tax reform in the United States increased the labor force participation of single women with children [26]. Zhou Lian and Chen Ye were the first to introduce the double-difference method into China in 2005, and used relevant socio-economic data from 1999 to 2002 for 591 counties and county-level cities in seven provinces to demonstrate the policy effects of rural tax reform in China, proving that rural tax reform did have a considerable positive impact on the growth rate of farmers’ income [27]. Zheng Xinye, Wang Han, and Zhao Yizhuo estimated the impact of the “province directly administered counties” reform on economic growth using a double-difference method based on data from Henan Province, China, in 2011, demonstrating that the “province directly administered counties” policy increased the economic growth rate of the counties directly administered by 1.3 percentage points after isolating the impact of other factors on economic growth [28]. Moser and Voena in 2012 demonstrated, through the difference in differences method, that certain developing countries have implemented compulsory licensing without the consent of foreign patent owners, i.e., allowing domestic firms to produce with foreign patents ultimately promotes domestic inventions [29]. Stefano Clòa and Elena Fumagalli studied the causal effect of imbalanced price regulations on the volume of the energy imbalances by using a quasi-experimental change data in regulation in the Italian power system through the difference in differences model [30]. Based on this, we propose to scientifically and effectively evaluate the pilot construction of Climate Resilient Cities in China using the difference in differences model. In order to make up for the blank of the existing research on “Climate Resilient Cities construction evaluation.”

In addition, the measurement of urban climate resilience is also a concern of many scholars. The weighting of indicators is the key to the evaluation of Climate Resilience, and common methods include subjective and objective empowerment methods and the analytic hierarchy process. Objective empowerment methods include principal component analysis, average empowerment methods, and the weighted correlation coefficient method, slacks-based measure models, data envelopment analysis(DEA) model [31], impact pathway approach [32], super-efficiency DEA models, and fuzzy decision-making trial and evaluation laboratory(DEMATEL) method. However, subjective empowerment methods are often subject to the personal preferences and knowledge of the researcher and cannot guarantee scientific validity, and the objective empowerment methods mentioned above often omit important information due to their simple calculation methods, which makes it difficult to fully reflect the true level of regional Climate Resilience [2]. These methods are typically only suitable for calculations on large samples; thus, new methods must be explored for scientifically valid measurements of Climate Resilience across regions [33].

The grey system theory is a new approach developed by Professor Deng Julong in 1982 to study the “incomplete data, poor information uncertainty problem” [34], and has since been applied to the study of the “low data, poor information uncertainty problem.” Tan Xue-rui and Deng Julong, two scholars of the method transformation, proposed a “grey correlation analysis,” also known as the “multi-level grey system evaluation method” [35], that is, to the factors of the sample data as the basis, with grey correlation to describe the strength of the relationship between the factors and the sample for ranking. If the sample data reflecting the two factors change trend is basically the same, then the correlation between the two is considered to be greater, otherwise the correlation between the two is considered to be smaller [21]. Grey correlation analysis measures the degree of correlation between data based on the degree of similarity or difference in developmental trends between factors, essentially comparing the proximity of the geometry of a curve formed by a number of series to the geometry of a curve formed by an ideal (standard) series. The degree of relevance reflects the order of proximity of the evaluation object to the ideal (criterion), i.e., the order of superiority or inferiority of the evaluation object, where the object with the greatest grey relevance is the best [36].

Social functioning is highly complex and uncertain, and the various statistical data representing social functioning may be grey due to human or technical problems. The study of the environmental aspects has been carried out in a number of research areas, including the following: the assessment of the environmental impact of the development of the environment [21], the assessment of satisfaction [37], the assessment of ecological vulnerability [38], the evaluation of safety incidents [39], and the assessment of air pollution [40]. The use of a multi-level grey system evaluation method in measuring climate resilience can overcome the gaps in existing studies and provide a more scientifically valid assessment of the climate resilience of regions.

## 3. Data and Method

### 3.1. Data Sources

The specific content of “Introduction of Pilot Policies for Climate Resilient Cities in China” can be found on the relevant government websites [41].

Since the statistical data of each prefecture-level city have problems, such as large differences in statistical content and inconsistent statistical caliber, if we take all prefecture-level cities in China as the object of study, there would be many missing data and, therefore, we would be unable to analyze the information. Instead, we took 16 prefecture-level cities in Anhui Province, China, as the object of study.

Anhui Province is located in the Yangtze River Delta region of China, between longitude 114°54′–119°37′ East and latitude 29°41′–34°38′ North. Anhui Province belongs to the transition area between the warm temperate zone and subtropical zone in terms of climate, north of Huaihe River belongs to warm temperate zone semi-humid monsoon climate, south of Huaihe River belongs to sub-humid monsoon climate, and Anhui Province is located in the middle and low latitude. This is a region with a monsoon climate, precipitation with distinct seasonal changes, and meteorological conditions that are relatively complex and diverse. Therefore, using Anhui Province as a research sample can reflect the construction of climate-resilient cities well.

To ensure the accuracy and validity of the data, all the data used in this study were obtained from the Anhui Provincial Statistical Yearbook and the official website of the Anhui Provincial Bureau of Statistics [42], and all the variables are continuous variables. Since policy implementation generally has a lag time, endogeneity issues may arise if regression analysis of control variables against the climate resilience index is conducted for the same period. Therefore, this paper treats the climate resilience index as a lagged period.

### 3.2. Variables

The measurement of climate resilience has been one of the hot topics in the field of Climate Resilient Cities research, because the academic community of climate resilience measurement index system has not yet formed a unified standard. Considering the accessibility and availability of data, a paper by Xie Xinlu, Zheng Yan etc., established the index system as expanded and improved [21]. An indicator system of 24 specific indicators in 16 categories was established in five dimensions: the infrastructure construction index, water safety construction index, ecological space optimization index, urban emergency management capacity-building index and science, and technology and innovation action index. To a large extent, these indicators are consistent with the previous studies [19,20,21,22] and, thus, can effectively evaluate the level of regional climate resilience. We used the multi-level grey system evaluation method to measure the climate resilience capacity of each region.

(1) The Introduction of Multi-Level Grey System Evaluation Approach

The multi-level grey system evaluation approach is based on the following models:

(1) R=E×W

where: $R={\left[r_{1},r_{2},\cdots ,r_{m}\right]}^{T}$ is a vector of composite evaluation results for the mth evaluation object; and

(2) $$W={\left[w_{1},w_{2},\cdots ,w_{n}\right]}^{T}$$

is the vector of weightings assigned to the nth evaluation indicators, where, in

(3) $$\sum_{j=1}^{n}w_{j}=1,$$

E is a matrix of judgements for each indicator:

(4) $$E=\left[\begin{array}{cccc} \varepsilon_{1}\left(1\right) & \varepsilon_{1}\left(2\right) & \cdots & \varepsilon_{1}\left(n\right)\\ \varepsilon_{2}\left(1\right) & \varepsilon_{2}\left(2\right) & \cdots & \varepsilon_{2}\left(n\right)\\ \cdots & \cdots & \cdots & \cdots \\ \varepsilon_{m}\left(1\right) & \varepsilon_{m}\left(2\right) & \cdots & \varepsilon_{m}\left(n\right) \end{array}\right]$$

$\varepsilon_{i}\left(k\right)$is the number of correlations between the best value of indicator k and indicator k for the ith scenario, and is then ranked according to the value of R.

(2) Analysis Steps of Multi-Level Grey System Evaluation Approach

The analytical steps of the multi-level grey system evaluation method are as follows:

Step 1: Identify the optimal set of indicators $\left(F^{*}\right)$

(5) $$F^{*}=[j_{1}^{*},\;j_{2}^{*},\;\cdots ,\;j_{n}^{*}]$$

where $j_{k}^{*}(k=1,2,\cdots ,n)$ is the optimal value for indicator k.

Step 2: Record the compared sequence in the correlation analysis as D:

(6) $$D=\left[\begin{array}{cccc} j_{1}^{1} & j_{2}^{1} & \cdots & j_{n}^{1}\\ j_{1}^{2} & j_{2}^{2} & \cdots & j_{n}^{2}\\ \cdots & \cdots & \cdots & \cdots \\ j_{1}^{m} & j_{1}^{m} & \cdots & j_{n}^{m} \end{array}\right]$$

where $j_{k}^{*}$ is the raw value of indicator k for the ith evaluation.

Step 3: Standardize the indicator values.

As the indicators are often of different scales and orders of magnitude, they cannot be compared directly, and the original indicators must be standardized to ensure the reliability of the results. We set the interval of change for indicator *k* to $\left[j_{k1},j_{k2}\right]$. $j_{k1}$ is the minimum value of indicator k across all programs. $j_{k2}$ is the maximum value of indicator k across all scenarios, and then the raw value can be converted to a dimensionless value using the following formula:

(7) $$C_{k}^{i}\in \left[0,1\right]$$

For positive indicators:

(8) $$C_{k}^{i}=\frac{j_{k}^{i}-j_{k1}}{j_{k2}-j_{k1}}$$

For negative indicators:

(9) $$C_{k}^{i}=\frac{j_{k2}-j_{k}^{i}}{j_{k2}-j_{k1}}\;i=1,2,\cdots ,m;\;k=1,2,\cdots ,n.$$

This converts the D matrix into the C matrix.

(10) $$C=\left[\begin{array}{cccc} C_{1}^{1} & C_{2}^{1} & \cdots & C_{n}^{1}\\ C_{1}^{2} & C_{2}^{2} & \cdots & C_{n}^{2}\\ \cdots & \cdots & \cdots & \cdots \\ C_{1}^{m} & C_{1}^{m} & \cdots & C_{n}^{m} \end{array}\right]$$

Step 4: Calculate the correlation coefficient.

According to the grey system theory, with $\left\{C^{*}\right\}=\left[C_{1}^{*},\;C_{2}^{*},\;\cdots ,\;C_{n}^{*},\;\right]$ as the reference series and $\left\{C^{*}\right\}=\left[C_{1}^{i},\;C_{2}^{*},\;\cdots ,\;C_{n}^{*},\;\right]$ as the series to be compared, the correlation analysis can be used to find the correlation between the best value of the k indicator of the ith scenario and the kth indicator of the $\varepsilon_{i}\left(k\right)$th scenario, namely,

(11) $$\varepsilon_{i}\left(k\right)=\frac{\min_{i}\min{}_{k}\left|C_{k}^{*}-C_{k}^{i}\right|+\rho \max_{i}\max{}_{k}\left|C_{k}^{*}-C_{k}^{i}\right|}{\left|C_{k}^{*}-C_{k}^{i}\right|+\rho \max_{i}\max{}_{k}\left|C_{k}^{*}-C_{k}^{i}\right|}$$

where $\varepsilon_{i}\left(k\right)$ is the number of correlations of i on indicator k, ρ is the discrimination coefficient to reduce the influence of extreme values on the calculation, and ρ∈[0,1] is generally taken as ρ=0.5.

Step 5: Calculate the relevance.

From $\varepsilon_{i}\left(k\right)$, we obtain E, so that the composite evaluation results in:

(12) R=E×W

(13) $$r_{i}=\sum_{k=1}^{n}W\left(k\right)\times \varepsilon_{i}\left(k\right)$$

A larger value of $r_{i}$ indicates that the ith evaluated object is closer to the ideal (standard) value, thus ranking the advantages and disadvantages [29].

The Climate Resilience indicator system and the weighting and direction of each indicator are shown in the Table 1.

**Figure 1:**
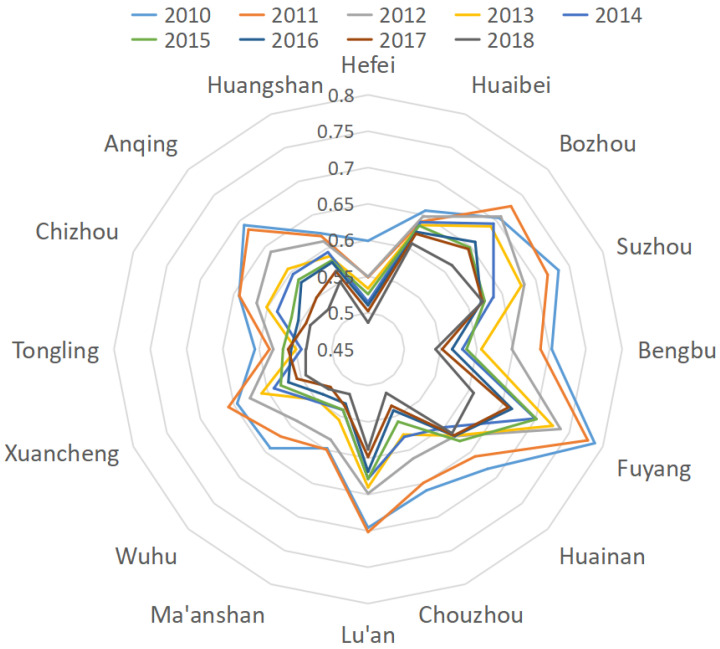
Radar chart of the Climate Resilience Index of each city (2010–2018).

The results of calculating the climate adaptability of prefecture-level cities in Anhui Province through the multi-level grey system evaluation method are shown in Figure 1.

To visualize the changes in the Climate Resilience Index (CRI) of 16 prefecture-level cities in Anhui Province from 2010 to 2018, the radar map of the CRI of each city (2010–2018) is plotted in this paper. From a temporal perspective, there was a general trend of decline in the Climate Resilience of the prefecture-level cities in Anhui province in recent years, which may be due to the severe ecological damage caused by major construction in recent years. From the perspective of geographical location, it can be seen from the figure that Fuyang, Bengbu, and Suzhou in the northern part of Anhui Province had a high Climate Resilience index, likely because the northern part of Anhui Province is relatively flat and less destructive to nature, with a relatively low level of urbanization and better ecological nourishment.

Hefei, Wuhu, and Ma’anshan in the central part of Anhui Province had a low Climate Resilience Index, likely because the central part of Anhui Province is reliant on the regional advantage of the radiation of the Yangtze River Delta for rapid economic development and the expansion of urban agglomeration to increase the climate risk. Huangshan and Tongling in the southern part of Anhui Province showed a low Climate Resilience Index, although the southern part of Anhui Province had a high level of economic development and invested more money in environmental and climate management. However, the southern part of Anhui Province is mainly mountainous and hilly, with complex geomorphology and frequent meteorological disasters, and thus the Climate Resilience index was low.

The remaining factors that may affect the local Climate Resilience are included in the regression model as control variables, including the GDP per capita (GDPPC), the density of population (DP), the urbanization (URB), the proportion of urban construction land (PUCL), the proportion of environmental fiscal expenditure (EFE), and industrial structure (IS).

### 3.3. Calculation Method

The calculation of these variables is shown in Table 2.

## 4. The Introduction of Difference in Differences Method

### 4.1. Difference in Differences Method

To study whether the pilot Cities’ Climate Resilience can be effectively improved, it is necessary to compare the changes in the Climate Resilience of the pilot areas before and after the implementation of the policy. However, there are many factors that affect a region’s Climate Resilience, such as the GDP per capita, the density of population, the urbanization, the proportion of urban construction land, the proportion of environmental fiscal expenditure, the industrial structure, and other factors, that have the potential to affect the regional climate adaptive capacity [43]. In addition, a region’s Climate Resilience can be affected by other macro-political, economic, and social factors.

For example, factors, such as reorganization of the environmental and emergency management sectors, personnel changes in local principal officials, and adjustments in economic development strategies may have an impact on the regional Climate Resilience. It is, therefore, problematic to judge the effectiveness of reforms solely on the basis of changes in the Climate Resilience after the pilot. The reason behind a region’s high Climate Resilience may not be pilot building, but may be due to macro factors. Thus, we introduced a difference in differences method in the process of studying the effects of pilot construction in Climate Resilient Cities.

The difference in differences model was set up by choosing a “pilot group” of areas that were included in the pilot program and a “control group” of areas that were not included in the pilot program, and by controlling for other factors, the difference between the pilot group and the control group after the policy occurred was compared to test the effect of the policy.

In this paper, the “Climate Resilience Index,” “Infrastructure Construction Index,” “Water Security Construction Index,” “Ecological Space Optimization Index,” “Urban Emergency Management Capacity Building Index,” and “Science and Technology Innovation Index” were used as the explanatory variables; the variable “climate resilience city construction pilot” $Pilot_{ij}$ was used to reflect whether the area of interest is a climate resilience city construction pilot or not, and the value of 1 indicates that the area is a climate resilience city construction pilot, and the value of 0 means that the area is not a climate resilience city construction pilot; according to the “Notice on Pilot Work of Climate Resilience City Construction,” the pilot group includes Hefei City and Huaibei City, and the control group includes Bozhou City, Suizhou City, Bengbu City, Fuyang City, Huainan City, Chuzhou City, Lu’an City, Ma’anshan City, Wuhu City, Xuancheng City, Tongling City, Chizhou City, Anqing City, and Huangshan City.

The variable “pilot time” $Time_{ij}$ was used to reflect the process of the pilot, and the value of 1 was taken in the year when the pilot construction policy of “climate resilience city” was implemented (2017) and thereafter, and 0 was taken otherwise. To test the effect of the pilot, we set up an interactive item, the “Climate Adaptive City Pilot Policy” $Did_{ij}$ to measure the pilot effect. In this way, we divided the sample into four groups: a pilot group before the pilot began ($Pilot_{ij}=1,\;Time_{ij}=0$), a pilot group after the pilot began ($Pilot_{ij}=1,\;Time_{ij}=1$), a control group before the pilot began ($Pilot_{ij}=0,\;Time_{ij}=0$), and a control group after the pilot began ($Pilot_{ij}=0,\;Time_{ij}=1$). The difference in differences model is thus:

(14) $$Y_{ij}=\beta_{0}+\beta_{1}Pilot_{ij}+\delta_{0}Time_{ij}+\delta_{1}Did_{ij}+\varepsilon_{ij}$$

Of these, $\beta_{1}$ reflects the differences between the pilot and control groups, $\delta_{0}$ reflects the impact of the pilot time on the pilot and control groups, and $\delta_{1}$ reflects the effects of the pilot. This is explained as follows:

For the control group, i.e., $Pilot_{ij}=0$, the above difference in differences model is noted as

(15) $$Y_{ij}=\left\{ \begin{array}{l} \beta_{0}\\ \beta_{0}+\delta_{0} \end{array}\right.\frac{Time_{ij}=0}{Time_{ij}=1}$$

Therefore, before and after the pilot, the change in the Climate Resilience index for the control group was $\delta_{0}$, with $\delta_{0}$ reflecting the impact of macro-political, economic, and social factors on the region’s Climate Resilience.

For the pilot group, i.e., $Pilot_{ij}=1$, the above difference in differences model is noted as

(16) $$Y_{ij}=\left\{ \begin{array}{l} \beta_{1}+\beta_{0}\\ \beta_{0}+\beta_{1}+\delta_{0}+\delta_{1} \end{array}\right.\frac{Time_{ij}=0}{Time_{ij}=1}$$

Thus, the change in the Climate Resilience Index for the pilot cities of the Climate Resilient Cities before and after the pilot is $\delta_{0}+\delta_{1}$, and so the net effect of the Cities for Climate Resilient Cities policy is $\delta_{0}+\delta_{1}-\delta_{0}=\delta_{1}$, which is the coefficient of, $Did_{ij}$, the interaction term of the variables $Pilot_{ij}$ and $Time_{ij}$. The sign for $\delta_{1}$ is positive if the Climate Resilient Cities Pilot policy contributes to the regional Climate Resilience and is negative if $\delta_{1}$ does not. The model provides a more accurate estimate of the impact of the Pilot Cities for the Climate Resilience policy on the regional Climate Resilience through a difference in differences process that allows for regional macro-political, economic, and social factors [36].

### 4.2. Difference in Differences Model Applicability Tests

While the difference in differences model is an effective tool for policy evaluation, the method is subject to the following stringent conditions for use.

**Premise** **1.** *“Common Trend (CT) Hypothesis,” i.e., whether there is the same trend in the Climate Resilience Index before and after the pilot in the experimental and control groups.*

The premise of the DID model assumes that, despite the differences between the pilot and control groups, the differences between the pilot and control groups are considered fixed as long as the trends prior to the pilot policy are consistent, and that the control group is a suitable “experimental” control group for the pilot group [36].

**Figure 2:**
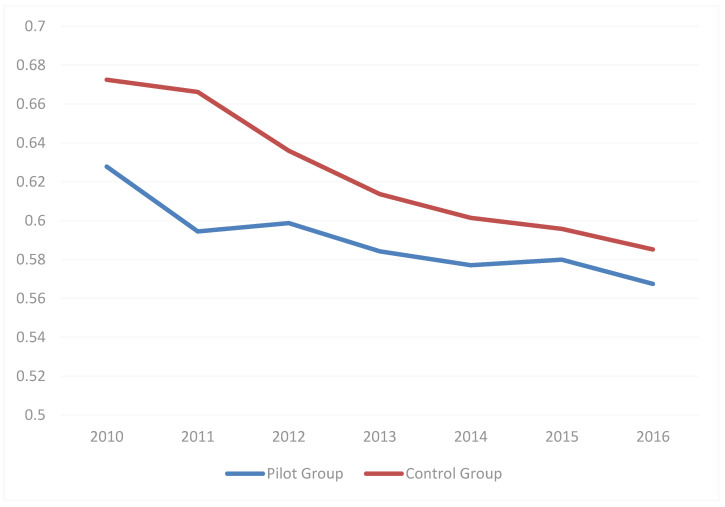
Climate Resilience Index for the pilot and control croups (2010–2016).

The DID model uses the “common trend (CT) assumption” in the premise, that is, the pilot and control groups are required to have the same trend in the Climate Resilience Index before the pilot construction; therefore, we selected the pilot and control groups of the historical mean value of Climate Resilience Index to plot the line graph. From the Figure 2, we can see that the pilot and control groups have the same trend.

**Premise** **2.** *Is the Selection of “Climate Resilient Cities” Pilots Random?*

In the process of policy evaluation, endogenous problems can easily arise. For example, in the case of pilot cities for Climate Resilient Cities, a city may be selected as a pilot city for Climate Resilient Cities because of its good climate endowment, thus, creating a reciprocal endogenous problem.

The National Development and Reform Commission (NDRC) and the Ministry of Housing and Urban–Rural Development (MOHURD) issued the Notice on Pilot Work for Climate Resilient Cities, which stated that the selection of pilot projects for Climate Resilient Cities are based on a comprehensive consideration of climate type, regional characteristics, stage of development, and work basis. To avoid endogeneity problems, this study used whether or not a city is a Climate Resilient City building pilot $Pilot_{ij}$ as the dependent variable and the Climate Resilience Index as the independent variable, and we included control variables in the model and used a logit model to test the selection criteria for the Climate Resilient city building pilot. The regression results are shown in Table 3.

Logit model regression shows that a region’s Climate Resilience Index is not necessarily related to whether it is selected as a pilot for climate resilient city building; thus, the selection of the pilot is exogenous.

## 5. Regression Models and Regression Results

### 5.1. Regression Model

Based on the results discussed earlier, to better identify the impacts of the Pilot Cities for Climate Resilient Cities policy, the study also controlled for GDP per capita, the density of population, the urbanization, the proportion of urban construction land, the proportion of environmental fiscal expenditure, the industrial structure, and other factors. The total measurement model for this study is therefore:

(17) $$Y_{ij}=\beta_{0}+\beta_{1}Pilot_{ij}+\delta Time_{0}{}_{ij}+\delta_{1}Did_{ij}+\beta_{2}GDPPC_{ij}+\beta_{3}DP_{ij}+\beta_{4}URB_{ij}+\beta_{5}PUCL_{ij}+\beta_{6}EFE_{ij}+\beta_{7}IS_{ij}+\varepsilon_{ij}$$

### 5.2. Descriptive Statistics

The descriptive statistics of all the variables involved in this paper are shown in Table 4.

### 5.3. Difference-in-Differences Model

To explore the impact of pilot construction policies on the regional Climate Resilience, this paper took the “Climate Resilience Index,” “Infrastructure Construction Index,” “Water Security Construction Index,” “Ecological Space Optimization Index,” “Urban Emergency Management Capacity Building Index,” and “Science and Technology Innovation Index” as the explanatory variables respectively, and the following six regression models were constructed using the difference in differences method. The specific regression model is shown in Table 5.

## 6. Discussion

The results of model 1, which was designed to investigate the impact of the pilot construction policy on regional Climate Resilience, showed that after controlling for the GDP per capita, population density, urbanization level, urban construction land share, fiscal expenditure on environmental protection, and industrial structure, the estimated impact of the pilot construction policy for Climate Resilient cities was 0.04, which was significantly positive at the 5% level, indicating that the pilot construction policy for Climate Resilient cities had a significant contribution to regional Climate Resilience, i.e., the pilot cities had a 4% higher Climate Resilience compared to the cities without pilot construction. In addition, the level of urbanization had a negative influence on the regional climate adaptation capacity—the higher the level of urbanization, the worse the climate adaptation capacity of the city, and the industrial structure had a positive influence on regional climate adaptation capacity—the higher the proportion of secondary industries, the better the climate adaptation capacity of the city.

Model 2 was designed to explore the impact of the Climate Resilient Cities pilot building policy on the regional infrastructure development. The results of Model 2 show that the estimate of the impact of the pilot construction policy for Climate Resilient Cities was 0.047 when all control variables were included in the model, which was significantly positive at the 1% level, indicating that the impact of the pilot construction policy for Climate Resilient Cities on regional level of infrastructure development had a significant catalytic effect, i.e., the pilot cities’ level of infrastructure development was 4.7% higher. In addition, the level of urbanization and the proportion of fiscal expenditure on environmental protection had an impact on the regional capacity-building for an infrastructure negative impact, and the industrial structure on the region level of infrastructure development had a positive effect—the higher the share of secondary industry, the higher the city’s level of infrastructure development.

Model 3 was designed to explore the impact of the Climate Resilient Cities pilot building policy on regional water security construction. The results of Model 3 showed an estimated impact of 0.008 when all control variables were included in the model; however, the impact is not significant, suggesting no impact of Climate Resilient Cities pilot construction policies on regional water security construction. While the level of construction of water security had a catalytic effect, it was not significant. In addition, the level of urbanization and the proportion of fiscal expenditure on environmental protection had an impact on the regional level of water security construction negative impact, and the industrial structure had a positive effect on the region level of water security construction—the higher the share of secondary industry, the higher the city’s level of water security construction.

Model 4 was designed to explore the impact of the Climate Resilient Cities pilot building policy on the regional ecological space optimization. The results of Model 4 showed that the estimate of the impact of the pilot construction policy for Climate Resilient Cities was 0.08 when all control variables were included in the model, which was significantly positive at the 5% level, indicating that the impact of the pilot construction policy for Climate Resilient Cities on the regional level of ecological space optimization had a significant catalytic effect, i.e., the pilot cities’ level of ecological space optimization was 8% higher. In addition, the level of urbanization had a significant negative impact on the regional level of ecological space optimization, and the share of fiscal expenditure on environmental protection and the structure of industry had a negative impact on the regional level of ecological space optimization with a positive effect—the higher the proportion of financial expenditure on environmental protection and the higher the proportion of secondary industries, the more the city’s level of ecological space optimization.

Model 5 was designed to explore the impact of Climate Resilient City pilot building policies on the capacity-building for urban emergency management. The results of Model 5 showed an estimated impact of 0.02 for the impact of the pilot construction policy for Climate Resilient Cities when all control variables were included in the model, but the impact was not significant, suggesting that, while the impact of the pilot construction policy for Climate Resilient Cities had a catalytic effect on the capacity building for urban emergency management, it was not significant. In addition, the level of urbanization, the share of financial expenditure on environmental protection, and the structure of industry all had a significant impact on the capacity building for urban emergency management with a significant negative impact. The higher the level of urbanization, the higher the proportion of financial expenditure on environmental protection, and the higher the proportion of secondary industries, the more the city’s level of emergency management capacity.

Model 6 was designed to explore the impact of the Climate Resilient Cities pilot building policy on regional science, technology, and innovation. The results of Model 6 showed that the estimated impact of the Climate Resilient Cities pilot construction policy was 0.046 when all control variables were included in the model, but the impact was not significant, indicating that while the impact of the Climate Resilient cities pilot construction policy on the urban STI had a catalytic effect, it was not significant. In addition, the impact of the GDP per capita and level of urbanization on the significant negative impact of urban STI financial expenditures on environmental protection as a percentage of industrial structure vs. urban STI had a significant positive impact.

## 7. Conclusions

In this paper, we use difference in differences model to evaluate the construction of the Climate Resilient Cities. The pilot policies of the “Climate Resilient Cities” showed a significant contribution to the regional climate resilience, and, after isolating the impact of other factors on the regional climate resilience, the pilot policies of the “Climate Resilient Cities” increased the climate resilience of the pilot cities by four percentage points. The pilot policies of the “Climate Resilient Cities” had a significant contribution to the urban infrastructure development and ecological space optimization, as well as non-significant impacts to the urban water security, emergency management capacity-building, and science and technology innovation initiatives.

This paper also has certain limitations: the first is that because of the statistical data lag, currently only data up to 2018 are available, and data for 2019 and beyond are not available; the second is that due to the inconsistency of statistical caliber across provinces, it is not possible to assess the climate adaptive capacity of all cities in the country. These issues await further research by other scholars.

## Figures and Tables

**Table 1:** Climate Resilience indicator system.

Level One Index	Level Two Index	Level Three Index	Level Four Index	Unit	Weight	Index Direction
Climate Resilience Index	Infrastructure Construction Index (1/5)	1.1. Transport Facilities	1.1.1. Urban Road Density	Kilometers Per Square Kilometer	1/5	+
			1.1.2. Number of Public Transport Means Per 10,000 People	Vehicles Per 10,000 Population	1/5	+
		1.2. Energy Facilities	1.2.1. Gas Penetration Rate	%	1/5	+
			1.2.2. Gas Network Density	Kilometers Per Square Kilometer	1/5	+
		1.3. Urban Renewal and Comprehensive Upgrading	1.3.1. Share of Urban Construction Projects in Total Investment in Urban Construction, in Terms of Reconstruction and Technical Improvements	%	1/5	+
	Water Security Construction Index (1/5)	2.1. Urban Water Supply Capacity	1.2.1. Daily Water Supply Per Capita	M^3^/Day	1/5	+
			1.2.2. Water access rate	%	1/5	+
		2.2. Urban Drainage capacity	2.2.1. Density of Urban Drainage Networks	Kilometers Per Square Kilometer	1/5	+
		2.3. Sewage Treatment capacity	2.3.1. Rate of Centralized Treatment in Urban Sewage Treatment Plants	%	1/5	+
		2.4. Water Resource Endowment	2.4.1. Water Resources Per Capita	10,000 M^3^/Person	1/5	+
	Ecological Space Optimization Index (1/5)	3.1. Afforestation	3.1.1. Total Forested Area as a Proportion of Urban Area	%	1/3	+
		3.2. Urban Greening	3.2.1. Green Space Coverage in Urban Built-up Areas	%	1/3	+
			3.2.2. Per Capita Green Space in Parks	Square Meter	1/3	+
	Urban Emergency Management Capacity Building Index (1/5)	4.1. Health Service System	4.1.1. Health Facility Staff Per 10,000 People	Persons Per 10,000	1/7	+
			4.1.2. Number of Beds in Health Facilities Per 10,000 People	Number of persons per 10,000	1/7	+
		4.2. Meteorological Security	4.2.1. Share of Expenditure on Land and Meteorological Services, etc.	%	1/7	+
		4.3. Integrated risk management capacity	4.3.1. Number of Environmental Emergencies	Times	1/7	-
			4.3.2. Number of Geological Disasters	Times	1/7	-
			4.3.3. Fire Rate Per 10,000 People	Times Per 10,000 people	1/7	-
			4.3.4. Traffic Accident Rate Per 10,000 People	Times Per 10,000 people	1/7	-
	Science and Technology Innovation Index (1/5)	5.1. Talent Training	5.1.1. Research and Experimental Development (R&D) Personnel as a Proportion of Total Population	%	1/4	+
		5.2. Research Institutions	5.2.1. Number of Research and Experimental Development (R&D) Research Institutions Per 10,000 People	Number of Persons Per 10,000	1/4	+
		5.3. Research Funding	5.3.1. Research and Experimental Development (R&D) Expenditure as a Proportion of Fiscal Expenditure	%	1/4	+
		5.4. Scientific and Technical Outputs	5.4.1. Patents Granted Per 10,000 People	Number of Persons Per 10,000	1/4	+

**Table 2:** The calculation method of the variables.

Category	Variable	Calculation Method	Unit
Dependent variable	Climate resilience index (CRI)	Multi-level grey system evaluation method	—
	Infrastructure Construction index (ICI)		—
	Water Safety Construction Index (WSCI)		—
	Ecological Space Optimization Index (ESOI)		—
	Urban Emergency Management Capability Index (UEMCI)		—
	Science and Technology Innovation Action Index (STIAI)		—
Control variable	GDP Per Capita (GDPPC)	GDP/total population	ten thousand yuan
	The Density of Population (DP)	Total population/land area of administrative area	10,000 people/km^2^
	Urbanization (URB)	Number of people in towns/total population	%
	The Proportion of Urban Construction Land (PUCL)	Urban built-up land area/administrative land area	%
	The Proportion of Environmental Fiscal Expenditure (EFE)	Financial expenditure on environmental protection/total financial expenditure	%
	Industrial Structure (IS)	Secondary industry output/gross output	%

**Table 3:** Binary choice model regression results.

Variable	Model
CRI	−16.313
	(38.182)
GDPPC	−4.826
	(3.047)
URB	0.695 *
	(0.421)
PUCL	65.869 *
	(37.097)
EFE	−25.248
	(62.063)
IS	0.171
	(0.13)
Constant	−32.605
	(27.104)
*N*	112
R^2^	0.748

Note: * denote significance at the 10% level, respectively.

**Table 4:** The descriptive statistics of the variables in the regression analysis.

Variable	Obs	Mean	Std. Dev.	Min	Max
GDPPC	144	3.881	2.299	0.832	12.3
DP	144	0.27	0.108	0.067	0.536
URB	144	51.45	11.685	29.1	78.68
PUCL	144	0.094	0.093	0.015	0.495
EFE	144	0.029	0.015	0.009	0.091
IS	144	50.717	9.382	34.9	74.7
CRI	144	0.608	0.06	0.487	0.788
ICI	144	0.564	0.054	0.425	0.719
WSCI	144	0.619	0.066	0.49	0.804
ESOI	144	0.602	0.091	0.413	0.943
UEMCI	144	0.468	0.042	0.373	0.602
STIAI	144	0.788	0.144	0.355	0.99
Pilot	144	0.125	0.332	0	1
Time	144	0.222	0.417	0	1
Did	144	0.028	0.165	0	1

**Table 5:** The difference in differences model.

Variable	Model 1	Model 2	Model 3	Model 4	Model 5	Model 6
	CRI	ICI	WSCI	ESOI	UEMCI	STIAI
Pilot	0.006	0.009	0.084 **	−0.007	0.027	−0.101
	(0.029)	(0.035)	(0.039)	(0.067)	(0.021)	(0.067)
Time	−0.026 ***	−0.045 ***	0.005	−0.034 **	−0.035 ***	−0.019
	(0.007)	(0.007)	(0.008)	(0.015)	(0.008)	(0.014)
Did	0.04 **	0.047 ***	0.008	0.08 **	0.02	0.046
	(0.016)	(0.017)	(0.019)	(0.035)	(0.017)	(0.031)
GDPPC	−0.006	−0.004	−0.001	0.003	0.005	−0.031 ***
	(0.004)	(0.005)	(0.005)	(0.01)	(0.004)	(0.009)
DP	0.054	−0.037	0.023	0.181	−0.037	0.146
	(0.051)	(0.056)	(0.064)	(0.114)	(0.045)	(0.105)
URB	−0.004 ***	−0.003 ***	−0.004 ***	−0.005 **	−0.003 ***	−0.004 *
	(0.001)	(0.001)	(0.001)	(0.002)	(0.001)	(0.002)
PUCL	−0.017	0.193	−0.097	−0.223	0.019	0.085
	(0.075)	(0.082)	(0.093)	(0.167)	(0.067)	(0.152)
EFE	0.273	−0.103 **	−0.438 *	1.172 ***	−0.059	0.803 **
	(0.193)	(0.206)	(0.232)	(0.427)	(0.203)	(0.377)
IS	0.002 ***	0.001 **	0.003 ***	0.003 **	−0.001 **	0.005 ***
	(0.001)	(0.001)	(0.001)	(0.001)	(0.001)	(0.001)
Constant	0.699 ***	0.674 ***	0.649 ***	0.678 ***	0.677 ***	0.766 ***
	(0.043)	(0.048)	(0.054)	(0.096)	(0.036)	(0.089)
*N*	144	144	144	144	144	144
R^2^	0.642	0.446	0.347	0.167	0.467	0.609

Note: ***, **, and * denote significance at the 1%, 5%, and 10% levels, respectively.

## Data Availability

The published statistical data comes from websites (http://tjj.ah.gov.cn/).
